# Prevalence and personal attitudes towards tobacco smoking among Palestinian healthcare professionals: a cross-sectional study

**DOI:** 10.1186/s13722-018-0119-z

**Published:** 2018-07-27

**Authors:** Isra Y. Mizher, Shahd I. Fawaqa, Waleed M. Sweileh

**Affiliations:** 1 College of Medicine and Health Sciences, An-Najah National University, Nablus, 44839 Palestine; 2 Department of Physiology, Pharmacology, and Toxicology, College of Medicine and Health Sciences, An-Najah National University, Nablus, 44839 Palestine

## Abstract

**Background:**

Little is known about tobacco smoking behaviors of healthcare professionals in the Middle East where stress conditions are high and tobacco smoking regulations are either absent or loose. The objective of this study was to identify the prevalence of and attitudes toward tobacco smoking among healthcare professionals.

**Methods:**

Trained senior medical students conducted a cross–sectional survey study in all governmental and non-governmental hospitals in Nablus city (Palestine) using a self-administered questionnaire containing both open-and closed-ended questions.

**Results:**

In total, 708 healthcare professionals participated in the study. The mean age of the participants was 31.4 ± 9.6 years. Forty-five (6.4%) participants were ex-smokers, 419 (59.2%) were never smokers, and 244 (34.5%) were current tobacco smokers. One hundred and forty-two (58.2%) tobacco smokers reported that they smoke inside the hospital and 119 (48.8%) reported that they think of quitting smoking. Univariate analysis indicated that age, gender, marital status, family history of tobacco smoking, country of graduation, and night shifts were significantly associated with tobacco smoking status. No significant difference (*p* = 0.156) in prevalence of tobacco smoking was found between physicians and other healthcare professionals. Binary logistic regression indicated that older age, male gender, and having a positive family history of smoking were significant predictors of being a current tobacco smoker. Non-smokers had significantly higher frequency of patient counseling than current smokers.

**Conclusion:**

Palestinian healthcare professionals have relatively higher prevalence of tobacco smoking compared to the general population. Urgent national intervention and strict implementation of “No Smoking Law” in health institutions and in public places are needed to root out this negative behavior.

## Background

Tobacco smoking is responsible for the death of nearly 7 million people yearly, 6 million of them are killed by direct tobacco use, while 890,000 are killed by second-hand smoking [1]. In addition, tobacco smoking is the second leading cause of cardiovascular diseases (CVD), contributing to approximately 12% of all cardiovascular disease deaths [2]. Tobacco smoking is also the single greatest avoidable risk factor for cancer mortality, as it has a relationship with many types of cancer [3, 4]. Furthermore, tobacco smoking plays a major role in physical fitness and survival [5].

The 2010 report by the *Palestinian Central Bureau of Statistics* (PCBS) revealed that 22.5% of adults (≥ 18 years old) were current tobacco smokers [6]. The report indicated that the percentage of male smokers reached 37.6 against 2.6% of female smokers. The report also stated that tobacco smoking plays a key role in the escalating numbers of lung cancers and childhood respiratory problems [7]. International reports also indicated that water tobacco smoking (hookah), which is a method of tobacco smoking, is gaining popularity where the highest prevalence of waterpipe smoking was in Lebanon (36.9%) followed by that in West Bank in the occupied Palestinian territory (32.7%) and Latvia (22.7%) [8]. Waterpipe smoking is common in the Middle East [9]. It is present in different flavors and is served in restaurants and coffee shops. The Council of Arab Ministers of Health called for banning smoking in all its forms in public and closed places, and prohibiting advertising and promotion of tobacco, its products and derivatives [10].

Physicians and other healthcare professionals should play a positive role in the prevention and treatment of tobacco smoking. Healthcare professionals who have direct contact with patients, should increase patients’ awareness about harms of smoking and should provide medical consultation for patients who want to quit smoking [11, 12]. Tobacco smoking among healthcare professionals negatively affects the attitude and impression of patients about healthcare professionals and negatively affects their willingness to quit smoking. Furthermore, it was found that physicians who smoke are less likely to advise their patients about smoking [13, 14] and their smoking status influence their patients’ response to quit smoking [15, 16]. Therefore, reducing the prevalence of tobacco smoking among healthcare professionals will effectively reduce its prevalence among the general population [17].

Combating tobacco smoking among healthcare professionals requires availability of data on the prevalence of this habit among this leading group of people. However, data on the prevalence of and attitudes toward tobacco smoking among healthcare professionals are lacking which negatively affects the ability of policy makers to develop national plans to ban tobacco smoking. Therefore, the general aim of the current study was to determine the prevalence of and attitudes toward tobacco smoking among healthcare professionals. The aim of this study is in accordance with World Health Organization (WHO) which recommends strengthening tobacco smoking surveillance and monitoring among various population categories and age groups [18, 19]. The findings of the current study helps the Palestinian legislative bodies to develop interventional strategies to minimize the health effects of tobacco smoking.

## Methods

### Study design and study population

This was a cross-sectional study that targeted healthcare professionals in two governmental and five non-governmental hospitals in the city of Nablus. The number of healthcare professionals in Nablus city is estimated to be less than 2000 while the estimated number of healthcare professionals in the selected seven hospitals is estimated to be 800. None of the hospitals included in the study had a clear, written, and implemented no-smoking policy. However, verbal recommendations and wall signs of “no smoking” are loosely adopted in these hospitals.

### Sample size

Sample size was calculated using the Raosoft sample size calculator [20]. The sample size was calculated by anticipating at least 20% prevalence of tobacco smoking with *z* = 1.96 for a 95% confidence level, margin of error of 5%, and a response rate of 50%. The minimum sample size required to estimate a population parameters was estimated as 323. The study was conducted from September 01, 2017 to November 01, 2017.

### Recruitment of participants

Two senior medical students visited the target hospitals three times weekly (Sunday, Tuesday, and Thursday) during the study period. The study questionnaire was distributed to healthcare professionals in all hospital departments on Sundays and Tuesdays and the filled questionnaire were collected back on Thursdays. Healthcare professionals were asked to fill in the questionnaire during working hours in their workplace. Completing the questionnaire took an average of 10 min. The participants were assured of the confidentiality of the information that they provided.

### The study tool

A self-administered questionnaire was developed for the purpose of this study. The questionnaire was developed based on previously published studies [21, 22]. The questionnaire consisted of three parts. The first part was about demographic characteristics of the participants, night shifts, family history of smoking, and their attitudes in dealing with patients who smoke. The first part included several questions about the role of the healthcare provider in advising patients about smoking and explaining its health hazards. The second part included questions to current tobacco smokers while the third part included questions to ex-smokers. Current smokers were asked about forms of tobacco smoking they use (cigarettes, waterpipe, or both), at what age they started smoking, for how long, and the quantity they smoke by day or week. Current smokers were also asked if they smoke inside the hospital and weather stressful conditions (yes, no) inside the hospital increase their tendency to smoke. Current smokers were also asked if they think of quitting smoking and by which means. Ex-smokers were asked about the age and the reason (medical, financial, other) for quitting smoking. In the current study, definitions of current and former cigarette smokers were obtained the Centre for Disease Control and Prevention guidelines [23] while waterpipe smokers were defined as in the study of Bahrain [22].

### Statistical analysis

Data collected were coded and entered into Statistical Package for Social Sciences (IBM SPSS statistics; version 21; Armonk, NY: IBM Corporation program. The main outcome variable was prevalence of current tobacco smoking expressed as a proportion (%). Most variables, such as gender, marital status, place of study, night shifts, place of living were entered as dichotomous variables (0; 1). Age variable was divided into four categories. Smoking status was divided into three categories: current smokers, never smokers, and ex-smokers. Descriptive categorical variables were expressed as proportion while descriptive continuous variables were expressed as mean ± standard deviation (SD) and/or medians and interquartile range (25th quartile–75th quartile). Statistical analysis for the association of various variables with smoking status was carried out using Chi square test using a significance level of less than 5%. Variables that showed significant association with smoking status in univariate analysis were entered into binary logistic regression to find significant predictors of smoking status. For the part pertaining to the attitudes of healthcare professionals towards smoking in clinical settings, the frequency of “yes” and “no” answers were calculated and statistically tested using Chi square test. Finally, for the ex-smokers, analysis was carried out separately because this category turned out to be small and because it is a unique category compared with current smokers and never smokers.

### Ethical consideration

Ethical approval was obtained from NNU-IRB committee, the Palestinian Ministry of Health (MOH), and each hospital’s medical director. A verbal consent was taken from each participant before filling in the questionnaire.

## Results

Of the 800 questionnaires that were distributed, a total of 708 were completed and returned to the researchers. The response rate was 88.5%. The mean ± SD of the age of the participants was 31.4 ± 9.6 years while the median (25th quartile–75th quartile) age was 28 [Q1–Q3 = 25–35] years. The sample included 387 (54.7%) males and 321 (45.3%) females. Forty-five (6.4%) were ex-smokers and this category of participants was analyzed separately to avoid any bias in data analysis and interpretation. The remaining 663 participants were either never smokers (419; 59.2%) or current smokers (244; 34.5%). Data regarding current smokers were analyzed in terms of socio-demographic characteristics, predictors of being a current smoker, types of smoking, and attitudes toward smoking habits in clinical settings.

### Smoking behaviors

When asked about how long they have been smoking, 50% reported that they have been smoking for at least 9 years. The mean ± SD of the starting age of smoking among current smokers was 21.1 ± 5.1 years. One hundred and forty-two (58.2%) current smokers reported that they smoke inside the hospital and 137 (56.1%) reported doing so because of the stressful conditions inside the hospital. When asked about where they smoke inside the hospital, the majority reported that they do so in hospital corridors or inside the medical staff rooms or food court. Sixty-eight (27.9%) of current smokers reported feeling embarrassed to smoke in the presence of patients and 170 (69.7%) reported that they wish they were not smokers. Only 5 (2.0%) reported that they would smoke in close distance to patients. Approximately half (119; 48.8%) of the current smokers reported that they think of quitting smoking using nicotine patches (30; 25.2%) or by strong will (89; 74.8%). When asked about why they think of quitting smoking, 87 (35.7%) cited economic reasons while the remaining stated health reasons. More than half (126; 51.9%) of current smokers reported smoking cigarettes only while the remaining (48.1%) reported smoking both cigarettes and waterpipe.

### Current versus never-smokers

Never and current smokers included 192 (29.0%) physicians and 471 (71%) nurses and other healthcare professionals. The non-physician group include 325 (49.0%) nurses while the remaining were medical laboratory technologists, pharmacists, and medical technicians. One hundred and twenty-three (18.6%) physicians were specialists. Socio-demographic characteristics of current smokers and never-smokers are shown in Table 1. The majority (363; 54.8%) of participants were in the age category of 25–34.9 years. Younger age category (< 25 years) had significantly lower odds [OR = 0.231; 95% CI (0.124–0.427)] of being smokers compared to older age category (> 45 years). The participants included 349 males and 314 females. Male healthcare professionals had significantly higher odds of being smokers than female healthcare professionals [OR = 7.099, 95% CI (4.889–10.309)]. The majority of participants were unmarried (383; 57%). Married healthcare professionals had significantly lower odds of being smokers than unmarried health professionals [OR = 0.685; 95% CI (0.496–0.948)]. More than half of the participants (358; 54%) had a family history of at least one of their parents being smoker. Healthcare professionals with positive family history of smoking had significantly higher odds of being smokers [OR = 2.446, 95% CI (1.759–3.401)]. When asked about place of study, the majority of participants were graduates of local Palestinian Universities (522, 78%). Healthcare professionals who studied in local universities had significantly lower odds of being smokers than health professionals who studied abroad [OR = 0.454, 95% CI (0.311–0.662)]. Approximately two-thirds of the participants reported having night shifts or having on-call shifts. Healthcare professional who had night or on-call shifts had significantly higher odds of being a smoker than those who do not [OR = 1.511, 95% CI (1.085–2.106)]. No significant difference in frequency of smoking was found between physicians and other healthcare professionals (*p* = 0.156). Similarly, both place of work and living address were not significantly associated with the status of smoking.

**Table 1:** Sociodemographic characteristics of current and never smokers

Variable	Total *N* = 663	Current smokers *N* = 244	Never smokers *N* = 419	*p* value
Age (years)				0.000
< 25	141 (21.27%)	27 (19.15%)	114 (80.85%)	
25–34.9	363 (54.75%)	144 (39.69%)	219 (60.33%)	
35–44.9	84 (12.69%)	35 (41.69%)	49 (58.33%)	
≥ 45	75 (11.31%)	38 (50.67%)	37 (49.33%)	
Gender				0.000
Male	349 (52.64%)	196 (56.16%)	153 (43.84%)	
Female	314 (47.37%)	48 (15.29%)	266 (84.71%)	
Marital status				0.022
Married	280 (42.23%)	89 (31.79%)	191 (68.21%)	
Unmarried	383 (57.77%)	155 (40.47%)	228 (59.53%)	
Living address				0.770
Urban	332 (50.07%)	124 (37.34%)	208 (62.65%)	
Suburban	331 (49.92%)	120 (36.25%)	211 (63.74%)	
Family history of smoking				0.000
Positive	358 (54.00%)	165 (46.10%)	193 (53.91%)	
Negative	305 (46.00%)	79 (25.90%)	226 (74.10%)	
Occupation				0.156
Physicians	192 (29.00%)	79 (41.14%)	113 (58.9%)	
Paramedics	471 (71.04%)	165 (35.03%)	306 (65%)	
Place of study				0.000
Inside the country	522 (78.73%)	171 (32.76%)	351 (67.24%)	
Abroad	141 (21.27%)	73 (51.77%)	68 (48.22%)	
Place of work				0.651
Governmental hospitals	313 (47.21%)	118 (37.70%)	195 (62.30%)	
Non-governmental hospitals	350 (52.79%)	126 (36.00%)	224 (64.00%)	
Night or on-call shifts				0.014
Yes	411 (62.00%)	166 (40.39%)	245 (59.61%)	
No	252 (38.00%)	78 (30.95%)	174 (69.04%)	

Significant variables in univariate analysis were entered in binary logistic regression using enter method to find the predictors of smoking status. The dependent variable was smoking status (current smoker vs. never smoker) while the independent variables were age category, gender, marital status, place of study, family history of smoking, and having night/on-call shifts. The results of binary logistic regression indicated that older age categories, male gender, and positive family history of smoking were significant predictors of a healthcare professional being a smoker (Table 2).

**Table 2:** Binary logistic regression for significant predictors of smoking status

Variable	*B*	*p* value	OR	95% CI for OR	
				Lower	Upper
Age category (< 25): reference					
Age category (25–34.9)	0.890	0.002	2.436	1.382	4.294
Age category (35–44.9)	0.938	0.019	2.556	1.164	5.612
Age category (> 45)	1.227	0.002	3.413	1.542	7.551
Gender (male) Gender (female): reference	2.000	0.000	7.390	4.867	11.223
Marital status (single) Marital status (married): reference	− 0.058	0.798	0.944	0.607	1.468
Place of graduation (local universities) Place of graduation (Abroad): reference	0.172	0.455	1.188	0.756	1.867
Night or on-call shifts No Yes (reference)	− 0.041	0.843	0.960	0.642	1.437
Family history of smoking Positive Negative (reference)	1.005	0.000	2.732	1.881	3.968
Constant	− 3.164	0.000	0.042		

*OR* odds ratio, *CI* confidence limit

The attitude of current smokers and never-smokers toward smoking was investigated. Approximately 92% of non-smokers were convinced that health professionals should advise patients to quit smoking while 81% of current smokers reported that they were convinced that they should advise patients to quit smoking (*p* < 0.001). Approximately 45% of non-smoker healthcare professionals reported that they always advise their patients to quit smoking while only 32% of current smokers reported doing so (*p* < 0.005). Furthermore, 116 (27.7%) of non-smokers reported that they follow up with their patients in smoking cessation while only 45 (18.4%) of current smokers said that they do so with their patients (*p* < 0.007). Approximately 89% of non-smokers reported that they endorse regulations to ban smoking of healthcare professionals in hospitals and clinical settings while 73% of smokers reported to agree on regulations that ban smoking of healthcare professionals in hospitals and clinical settings (*p* < 0.000). No significant difference was found between current smokers and non-smokers with regard to efforts and time spent with patients to explain negative health effects of smoking (*p* = 0.112) (Table 3).

**Table 3:** Counseling behaviors of current and never smokers

Question	Total	Never smokers	Current smokers	*p* value
Are you convinced that a healthcare worker should advise the patient to stop smoking?				
Yes	581 (87.63%)	384 (91.64%)	197 (80.73%)	0.000
No	82 (12.36%)	35 (8.35%)	47 (19.26%)	
Do you advise your patient to stop smoking?				
Always	266 (40.12%)	188 (44.87%)	78 (31.96%)	0.005
Sometimes	329 (49.62%)	192 (45.82%)	137 (56.15%)	
Never	68 (10.25%)	39 (9.30%)	29 (11.88%)	
Do you explain the negative health effects of smoking to your patient?				
Yes	550 (82.95%)	355 (84.73%)	195 (79.91%)	0.112
No	113 (17.04%)	64 (15.27%)	49 (20.08%)	
Do you follow up with your smoking patients if they reduce or quite smoking?				
Yes	161 (24.28%)	116 (27.68%)	45 (18.44%)	0.007
No	502 (75.71%)	303 (72.31%)	199 (81.56%)	
Have you recorded any cases that quitted smoking?				
High numbers	9 (1.35%)	8 (1.90%)	1 (0.40%)	0.193
Low numbers	184 (27.75%)	111 (26.49%)	73 (29.91%)	
Zero	470 (70.89%)	300 (71.59%)	170 (69.67%)	
Do you know if there are any policies that ban smoking of healthcare workers inside hospitals?				
Yes	263 (39.67%)	166 (39.62%)	97 (39.75%)	0.972
No	400 (60.33%)	253 (60.38%)	147 (60.24%)	
In your opinion, Is it a necessity to have policies that ban smoking and punish who smokes inside hospitals?				
Yes	550 (83.00%)	372 (88.87%)	178 (73.00%)	0.000
No	113 (17.04%)	47 (11.21%)	66 (27.04%)	
Total	663 (100%)	419 (100%)	244 (100%)	

### Characteristics of ex-smokers

Of the 708 participants, there were 45 healthcare professionals who identified themselves as ex-smokers. The majority of ex-smokers (26; 55.6%) stated that they were regular smokers for at least 5 years. Ex-smokers were 38 (84.4%) males and 7 (15.6%) females. Mean ± SD age of ex-smokers was 37 ± 12.1 years. Approximately half (51.1%) of ex-smokers had a positive family history of smoking. The majority of ex-smokers were married (77.8%). Ex-smokers included 19 (42.2%) physicians. When asked about the cause of quitting smoking, the majority (31; 68.9%) stated that they were concerned about the health consequences of smoking while the remaining stated economic reasons for quitting smoking. When asked about the method they used to quit smoking, only 6 (13.3%) stated using nicotine replacement therapy while the remaining stated strong will and sports as methods of quitting smoking.

## Discussion

In the current study, we investigated the prevalence of tobacco smoking among healthcare professionals working in governmental and non-governmental hospitals in Nablus district, north of Palestine. The current study showed that the prevalence of tobacco smoking among healthcare professionals was relatively higher than that reported in the general population as well as among the youth population in West Bank [6, 24]. Unfortunately, there is no previously published data about the prevalence of tobacco smoking among healthcare professionals in Palestine for comparative purposes. Therefore, the current study serves as a baseline data for future comparisons and for future interventional programs to combat tobacco smoking at the national level.

No doubt that occupational stress is one potential reason for the high prevalence of tobacco smoking among healthcare professionals compared to that of the general population [25, 26]. A second potential reason is the loose implementation of the Palestinian “No Smoking law” [27]. A third reason is the wrong belief that waterpipe smoking is less harmful than cigarette smoking [28]. A fourth potential reasons is the lack of well-trained experts in the treatment of nicotine addiction and in the delivery of smoking cessation therapy. The Palestinian Ministry of Health should invest in building capacities and starting specialized clinics for tobacco smoking cessation therapy to help in combating tobacco smoking. These factors should be considered in any tailored national intervention program to decrease the prevalence of tobacco smoking.

There are several studies about prevalence of tobacco smoking among healthcare professionals in the Middle East region. The findings of the current study was higher than that reported from Bahrain [22], Saudi Arabia [29, 30], and Oman [31], but lower than that reported from Jordan [32]. At the international level, the findings of the current study was closer to that reported from Central/Eastern Europe (37%) and higher than that in Africa (29%), Central and South America (25%), and Asia (17.5%) [33]. It was fortunate that only five of current smokers reported smoking in close distance to patients. This is in agreement with a study published from Croatia [26] but not in agreement with other studies [34]. Healthcare professionals who participated in our study are familiar with the harmful effects of smoking and passive smoking on patients, children, and pregnant women and tend to avoid smoking in front of patients.

The current study showed that the significant predictors of current tobacco smoking were older age, male gender, and positive family history of smoking. The findings of the current study regarding predictors of smoking status were in agreement with those published in other studies [35–37]. Gender differences in the prevalence of smoking are due to the conservative culture in the Middle East. Women who smoke are subjected to social stigma. Therefore, the data regarding prevalence of tobacco smoking among females might be inaccurate due to fear of females of social stigma. Despite this, interventional programs to combat smoking should focus on males and give women a participatory role in education and increasing awareness among males. The strong association between tobacco use and parental history of tobacco use indicates that smokers might have inherited this bad habit from their parents which make them victims of their living environment.

The current study showed that almost half of the current tobacco smokers use both cigarette smoking and waterpipe. The relationship between waterpipe tobacco smoking and cigarettes had been discussed is complex and showed cultural variations [38]. The availability of various flavors of waterpipe and the social acceptability of waterpipe are considered a precursor for future cigarette smoking [39]. A longitudinal smoking study carried out in Irbid (Jordan) suggested that waterpipe tobacco smoking may be an initial trigger to future cigarette smoking among never users [40]. A Canadian study argued that waterpipe smokers have higher prevalence of substance use than non-smokers [41]. A recent study from Palestine concluded that there is a high prevalence of waterpipe smoking that surpassed the prevalence of cigarette smoking [24]. The authors of the Palestinian study concluded that interventions to curb the practice of tobacco smoking among Palestinian youth should be tailored differently to waterpipe smoking and cigarette smoking. The common use of waterpipe smoking among healthcare professionals could be attributed to the increasing trend of waterpipe smoking among youth and university students [42]. It should be emphasized here that cigarette smoking is usually an individual and might be a hidden behavior. However, the waterpipe smoking is usually practiced within social groups and in public places which negatively affects the public image of healthcare professionals and negatively affects their abilities to provide clinical intervention in smoking cessation therapy.

The current study has few limitations. First, the study was not a national study. Only healthcare professionals in Nablus city participated in the study. It should emphasized here that different regions in Palestine might have different patterns and prevalence of smoking [24]. Second, the recruitment of the participants and the study setting might have created certain bias or underestimation of the prevalence of tobacco smoking among female healthcare professionals. Third, the tool used in this study was developed by the authors and not an internationally validated one. Fourth, the prevalence of tobacco smoking was not correlated with any laboratory or psychological measure of nicotine addiction. Future studies should include measures of nicotine addiction and correlate it with prevalence and attitude toward smoking. Fifth, the cross-sectional design of the study which limits the ability of the investigators to generalize the findings of the study or claim any causal relationship between demographic variables and smoking status.

## Conclusion

Our study indicated that the prevalence of tobacco smoking among healthcare professionals in hospitals is relatively higher than that reported elsewhere including the general population in Palestine. This finding could create a negative image about healthcare professionals who should behave as a model for disease prevention. We have also shown that male gender, parental history of tobacco smoking, and older age are significant predictors that need to be targeted in future plans to decrease prevalence of smoking. In Palestine, there is weak implementation of the “No Smoking Law” issued in 2005 [27] and it is hoped that the current findings signal a warning to health policy makers and legislative bodies to strengthen the implementation of the law and impose penalties for those who violate the law in health institutions. The ultimate goal is to formulate a national plan in which healthcare professionals can take the lead in both increasing awareness and in delivering appropriate smoking cessation therapy.

## Abbreviation

IRB: Institutional Review Board

## References

[1] World Health Organization (WHO); Tobacco (Fact sheet). http://www.who.int/mediacentre/factsheets/fs339/en/. 28 Jan 2018.

[2] World Health Organization (WHO); World No Tobacco Day, Tobacco and heart disease. http://www.who.int/mediacentre/events/2018/world-no-tobacco-day/en/. 28 Jan 2018.

[3] Motorykin O, Matzke MM, Waters KM, Massey Simonich SL (2013). Association of carcinogenic polycyclic aromatic hydrocarbon emissions and smoking with lung cancer mortality rates on a global scale. Environ Sci Technol.

[4] Islami F, Torre LA, Jemal A (2015). Global trends of lung cancer mortality and smoking prevalence. Transl Lung Cancer Res.

[5] West R (2017). Tobacco smoking: health impact, prevalence, correlates and interventions. Psychol Health.

[6] Palestinian Central Bureau of Statistics (PCBS): Palestinian Family Survey, 2010 (Final Report). In: Ramallah—State of Palestine; 2013.

[7] Palestinian Central Bureau of Statistics (PCBS) and the Ministry of Health (MoH): The Palestinian Central Bureau of Statistics (PCBS) and the Ministry of Health (MoH) are issuing a Press Release on the occasion of International Day of Giving up Smoking (Word No Tobacco Day) In: Rmallah—State of Palestine; 2012.

[8] Jawad M, Lee JT, Millett C (2015). Waterpipe tobacco smoking prevalence and correlates in 25 Eastern Mediterranean and Eastern European countries: cross-sectional analysis of the Global Youth Tobacco Survey. Nicotine Tob Res.

[9] Jaghbir M, Shreif S, Ahram M (2014). Pattern of cigarette and waterpipe smoking in the adult population of Jordan. East Mediterr Health J.

[10] Asharq Al-Awsat; Arab Countries Considering broad ban on smoking. https://aawsat.com/english/home/article/1191901/arab-countries-considering-broad-ban-smoking. 02 July 2018.

[11] Joshi V, Suchin V, Lim J (2010). Smoking cessation: barriers, motivators and the role of physicians—a survey of physicians and patients. Proc Singap Healthc.

[12] Sreedharan J, Muttappallymyalil J, Venkatramana M (2010). Nurses’ attitude and practice in providing tobacco cessation care to patients. J Prev Med Hyg.

[13] Abdullah AS, Guangmin N, Kaiyong H, Jing L, Yang L, Zhang Z, Winickoff JP (2018). Implementing tobacco control assistance in pediatric departments of chinese hospitals: a feasibility study. Pediatrics.

[14] Parna K, Rahu K, Rahu M (2005). Smoking habits and attitudes towards smoking among Estonian physicians. Public Health.

[15] Stead LF, Buitrago D, Preciado N, Sanchez G, Hartmann-Boyce J, Lancaster T (2013). Physician advice for smoking cessation. Cochrane Database Syst Rev.

[16] Meshefedjian GA, Gervais A, Tremblay M, Villeneuve D, O’Loughlin J (2010). Physician smoking status may influence cessation counseling practices. Can J Public Health.

[17] Shkedy YOFR, Mizrachi A (2013). Smoking habits among Israeli hospital doctors: a survey and historical review. Israel Med Assoc J IMAJ.

[18] World Health Organization (WHO): WHO Study Group on Tobacco Product Regulation (TobReg), Waterpipe tobacco smoking: health effects, research needs and recommended actions for regulators. In: Edited by ed. n; 2015.

[19] World Health Organization (WHO) (2013). Global action plan for the prevention and control of NCDs 2013–2020.

[20] Raosoft; Sample size calculator. http://www.raosoft.com/samplesize.html. 02 July 2018.

[21] Zinonos S, Zachariadou T, Zannetos S, Panayiotou AG, Georgiou A (2016). Smoking prevalence and associated risk factors among healthcare professionals in Nicosia general hospital, Cyprus: a cross-sectional study. Tob Induc Dis.

[22] Borgan SM, Jassim G, Marhoon ZA, Almuqamam MA, Ebrahim MA, Soliman PA (2014). Prevalence of tobacco smoking among health-care physicians in Bahrain. BMC Public Health.

[23] CDC (2009). State-specific secondhand smoke exposure and current cigarette smoking among adults—United States, 2008. MMUR?.

[24] Tucktuck M, Ghandour R, Abu-Rmeileh NME (2017). Waterpipe and cigarette tobacco smoking among Palestinian university students: a cross-sectional study. BMC Public Health.

[25] Ficarra MG, Gualano MR, Capizzi S, Siliquini R, Liguori G, Manzoli L, Briziarelli L, Parlato A, Cuccurullo P, Bucci R (2011). Tobacco use prevalence, knowledge and attitudes among Italian hospital healthcare professionals. Eur J Public Health.

[26] Juranić B, Rakošec Ž, Jakab J, Mikšić Š, Vuletić S, Ivandić M, Blažević I (2017). Prevalence, habits and personal attitudes towards smoking among health care professionals. J Occup Med Toxicol.

[27] Palestinian Legislative Council: Public Health Law. In: Ramallah—Palestine; 2005.

[28] Akl EA, Jawad M, Lam WY, Co CN, Obeid R, Irani J (2013). Motives, beliefs and attitudes towards waterpipe tobacco smoking: a systematic review. Harm Reduct J.

[29] Al-Haddad NS, Al-Habeeb TA, Abdelgadir MH, Al-Ghamdy YS, Qureshi NA (2003). Smoking patterns among primary health care attendees, Al-Qassim region, Saudi Arabia. East Mediterr Health J.

[30] Mahfouz AA, Shatoor AS, Al-Ghamdi BR, Hassanein MA, Nahar S, Farheen A, Gaballah II, Mohamed A, Rabie FM (2013). Tobacco use among health care workers in Southwestern Saudi Arabia. Biomed Res Int.

[31] Al-Lawati JA, Nooyi SC, Al-Lawati AM (2009). Knowledge, attitudes and prevalence of tobacco use among physicians and dentists in Oman. Ann Saudi Med.

[32] Shishani K, Nawafleh H, Jarrah S, Froelicher ES (2011). Smoking patterns among Jordanian health professionals: a study about the impediments to tobacco control in Jordan. Eur J Cardiovasc Nurs.

[33] Abdullah AS, Stillman FA, Yang L, Luo H, Zhang Z, Samet JM (2013). Tobacco use and smoking cessation practices among physicians in developing countries: a literature review (1987–2010). Int J Environ Res Public Health.

[34] Duaso M, Duncan D (2012). Health impact of smoking and smoking cessation strategies: current evidence. Br J Community Nurs.

[35] Stamatopoulou E, Stamatiou K, Voulioti S, Christopoulos G, Pantza E, Stamatopoulou A, Giannopoulos D (2014). Smoking behavior among nurses in rural greece. Workplace Health Saf.

[36] Lam TH, Jiang C, Chan YF, Chan SSC (2011). Smoking cessation intervention practices in Chinese physicians: do gender and smoking status matter?. Health Soc Care Community.

[37] Jiménez-Ruiz CA, Miranda JAR, Pinedo AR, Martinez EDH, Marquez FL, Cobos LP, Reina SS, Orive JIDG, Ramos PDL (2015). Prevalence of and attitudes towards smoking among Spanish health professionals. Respiration.

[38] Jawad M, Lee JT, Millett C (2014). The relationship between waterpipe and cigarette smoking in low and middle income countries: cross-sectional analysis of the global adult tobacco survey. PLoS ONE.

[39] Jensen PD, Cortes R, Engholm G, Kremers S, Gislum M (2010). Waterpipe use predicts progression to regular cigarette smoking among Danish youth. Subst Use Misuse.

[40] Mzayek F, Khader Y, Eissenberg T, Al Ali R, Ward KD, Maziak W (2012). Patterns of water-pipe and cigarette smoking initiation in schoolchildren: Irbid longitudinal smoking study. Nicotine Tob Res.

[41] Dugas E, Tremblay M, Low NC, Cournoyer D, O’Loughlin J (2010). Water-pipe smoking among North American youths. Pediatrics.

[42] Jawad M, McEwen A, McNeill A, Shahab L (2013). To what extent should waterpipe tobacco smoking become a public health priority?. Addiction.

